# Supplementary material for: Gli3 utilizes Hand2 to synergistically regulate tissue-specific transcriptional networks
Source: eLife. 2020 Oct 2;9:e56450. doi: 10.7554/eLife.56450 (PMC7556880; doi:10.7554/eLife.56450)
Supplement: Supplementary file 1. [file elife-56450-supp1.docx]

**Supplemental Table 1.** Differences in gene expression levels from conditional KO study

|  | *Gli2^f/f^;Gli3 ^f/f^;Wnt1-Cre* | | *Hand2 ^f/f^;Wnt1-Cre* | |
| --- | --- | --- | --- | --- |
| **Gene** | **Fold Change^a^** | **Significance^b^** | **Fold Change^a^** | **Significance^b^** |
| *Hand2* | -1.1 | n.s. | -8.1 | p<0.05 |
| *Gli1* | -3.9 | p<0.05 | -1.0 | n.s. |
| *Gli2* | -1.8 | p<0.05 | -1.1 | n.s. |
| *Gli3* | -2.2 | p<0.05 | -1.1 | n.s. |
| *Ptch1* | -1.8 | p<0.05 | -1.2 | n.s. |
| *Shh* | 1.7 | p<0.05 | 1.0 | n.s. |

^a^Relative to wild-type

^b^not significant; n.s.
